# Supplementary material for: Structure-property relationship of Co2MnSi thin films in response to He+-irradiation
Source: Sci Rep. 2019 Feb 26;9:2766. doi: 10.1038/s41598-019-39435-4 (PMC6391387; doi:10.1038/s41598-019-39435-4)
Supplement: Supplementary file 1 — SUPPLEMENTARY MATERIAL - Structure-property relationship of Co$_2$MnSi thin films in response to He$^+$-irradiation [file 41598_2019_39435_MOESM1_ESM.pdf]

SUPPLEMENTARY MATERIAL - Structure-property relationship of Co_2MnSi thin films in response to He^+-irradiation

Franziska Hammerath[1,*], Rantej Bali[2], René Hübner[2], Mira R. D. Brandt[1], Steven Rodan[1], Kay Potzger[2], Roman Böttger[2], Yuya Sakuraba[3], and Sabine Wurmehl[1,4]

[1]IFW-Dresden, Institute for Solid State Research, Helmholtzstraße 20, 01069 Dresden, Germany
[2]Helmholtz-Zentrum Dresden-Rossendorf, Institute of Ion Beam Physics and Materials Research, Bautzner Landstrasse 400, 01328 Dresden, Germany
[3]National Institute of Materials Science, Tsukuba, Ibaraki, Japan
[4]Institute of Solid State and Materials Physics, TU Dresden, 01062 Dresden, Germany
[*]corresponding author: f.hammerath@ifw-dresden.de

Structure types for Co_2MnSi relevant in the present work

The $L2_1$ type structure of Co_2MnSi (Strukturbericht designation, see figure 1(a), spacegroup $Fm\bar{3}m$, inorganic structure database (ICSD) notation Cu_2MnAl) may be visualized as a superstructure of the CsCl lattice, in which the lattice parameter of the CsCl lattice is doubled in all three dimensions. This leads to a cell consisting of eight cubes with one atom in each center. The 16 atoms in this supercell may be divided in four groups consisting of four atoms. The atoms in the center of each cube are the Co atoms [Wykhoff position ($8c$, $\frac{1}{4}\frac{1}{4}\frac{1}{4}$)], while the Mn ($4b$, $\frac{1}{2}\frac{1}{2}\frac{1}{2}$) and Si atoms ($4a$, 0 0 0) occupy the corners of the eight cubes. Each atom is face centered cubic ordered, leading to four interpenetrating face centered cubic lattices. The local environment of ^{59}Co consists of 4 Mn and 4 Si atoms, giving rise to one single resonance line in NMR. In case of small off-stoichiometries, additional lines at the lower or higher frequency side of the main line may show up (see, e.g.,[1–3]).

If the Co, Mn, and Si atoms are statistically distributed on all crystallographic sites, only one Wykhoff position ($2a$), different (local) symmetries, and the $A2$ type structure are obtained [$Im\bar{3}m$, ICSD notation tungsten (W) type[4], see figure 1(b)]. This random mixing of all atoms leads to a plethora of possible local first, next nearest, next-next nearest, etc. environments for ^{59}Co giving rise to one broad resonance line with a width of about 90 MHz consisting of many unresolved sublines that stem form the huge number of local environments[3,5–7].

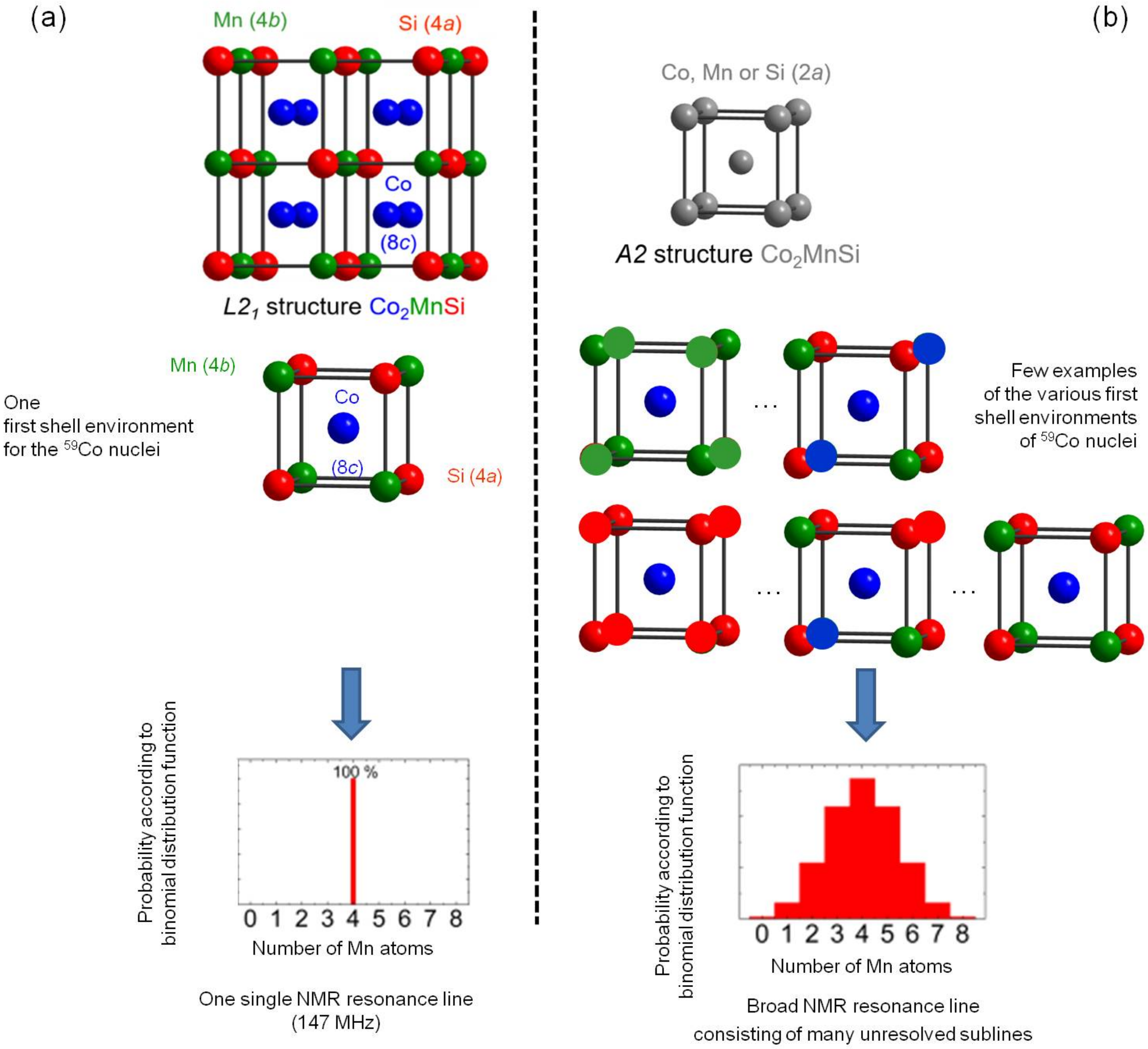

Figure 1. (Color online) (a) The $L2_1$ type structure of Co_2MnSi where all ^{59}Co nuclei have the same first shell environment (4Mn + 4 Si atoms), leading to one NMR resonance line observed at 147 MHz. (b) The $A2$ type structure is obtained if the Co, Mn, and Si atoms are statistically distributed on all crystallographic sites, leading to various types of local environments. Since each specific local environment has a specific NMR resonance frequency, one observes a broad resonance consisting of many unresolved sublines.

Simulated depth variation of the atomic displacements

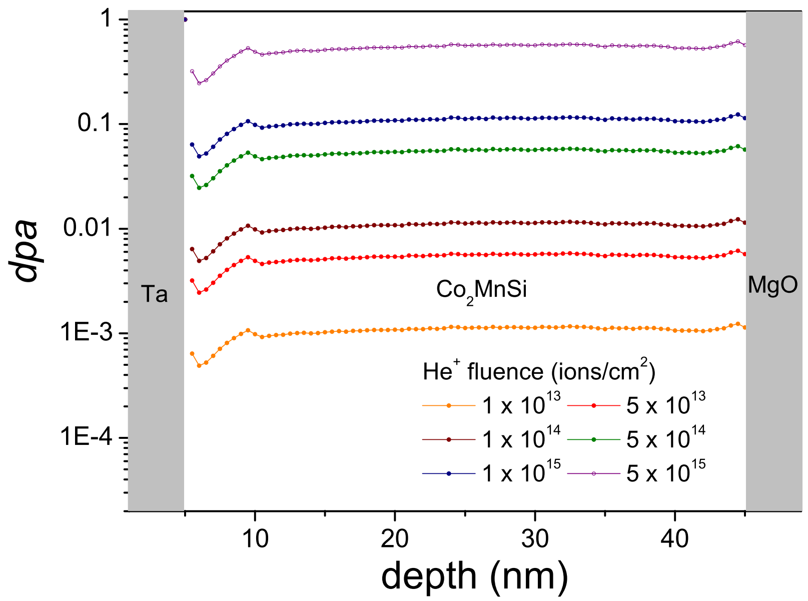

Figure 2. (Color online) Simulated depth variations of the displacements per atom (dpa) in the Co_2MnSi thin films for 15 keV He^+ ions of given fluence (see legend).

Off-stoichiometric bulk sample

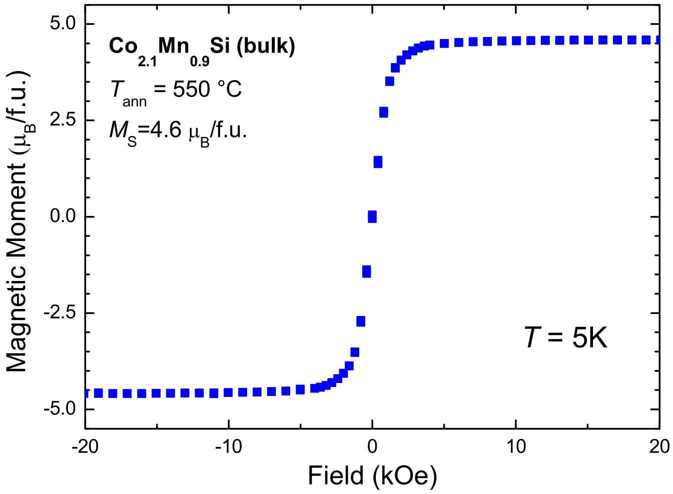

Figure 3. (Color online) Magnetization of an off-stoichiometric $Co_{2.1}Mn_{0.9}Si$ bulk sample measured at 5 K. The saturation magnetization amounts to 4.6 μ_B/f.u..

HRTEM of the non-irradiated reference sample

Fig. 4 shows cross-sectional HRTEM data for the non-irradiated reference sample (a) together with fast Fourier transformation (FFT) patterns at selected areas in the lower (b) and upper (c) part of the TEM image. For the analyzed regions of the non-irradiated reference sample, the obtained FFTs can be described with the $L2_1$ structure in [101] zone axis geometry, in nice agreement with our NMR results.

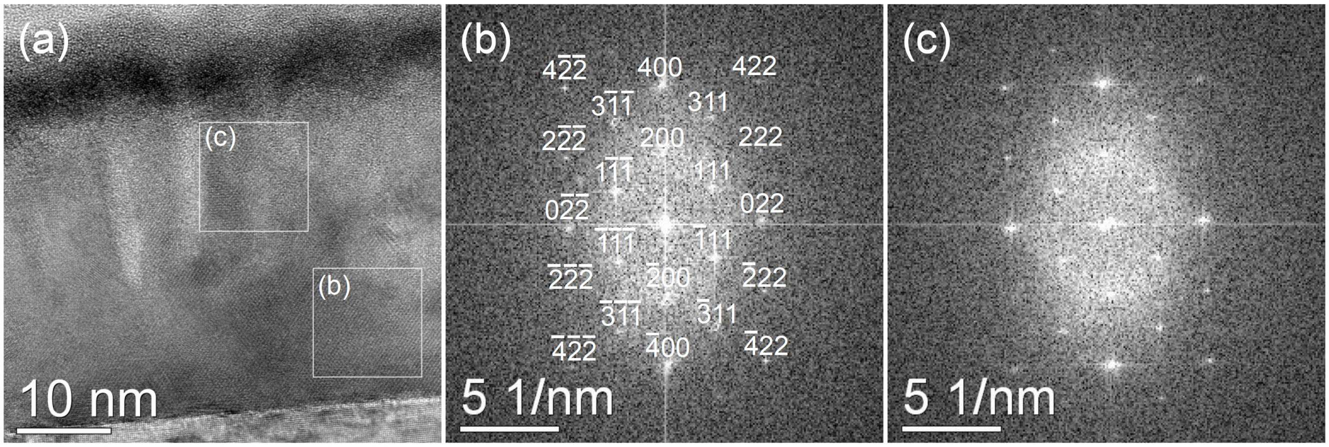

Figure 4. Cross-sectional HRTEM image (a) and corresponding fast Fourier transforms for the lower part (b) and upper part (c) of the non-irradiated reference sample, as obtained from the marked square areas.

Oxide inclusions

Fig. 5 shows a cross-sectional HAADF-STEM image of the sample irradiated with 5×10^{14} ions/cm^2. Besides inclusions in the upper part of the Co_2MnSi film which appear dark and, hence, point to a decreased mean atomic number, the Ta capping layer shows a two-fold gradation. While the near-surface part which appears light-grey is obviously completely oxidized, the lower part of the Ta film shows the oxidic behavior only directly above the dark inclusions. Hence, these inclusions are supposed to be oxidized Co_2MnSi. To validate this assumption, EDXS spectrum imaging was performed. The corresponding Co, Mn, Si, and O element distributions are shown in Figs. 5(b)-(e), respectively. Please note that the enhanced Si signal within the Ta capping layer region is not real, but caused by the superposition of the Si-$K_{\alpha1,2}$ peak (1740 eV) with the Ta-$M_{\alpha1}$ peak (1710 eV). According to the EDXS-based chemical analysis, the dark inclusions within the Co_2MnSi film show reduced cobalt and manganese signals and an enhanced oxygen content. Consequently, the Ta capping layer seems to be not thick enough to prevent partial Co_2MnSi oxidation. It should be mentioned that the non-irradiated sample shows a comparable behavior with oxidized inclusions in the upper part of the Co_2MnSi film.

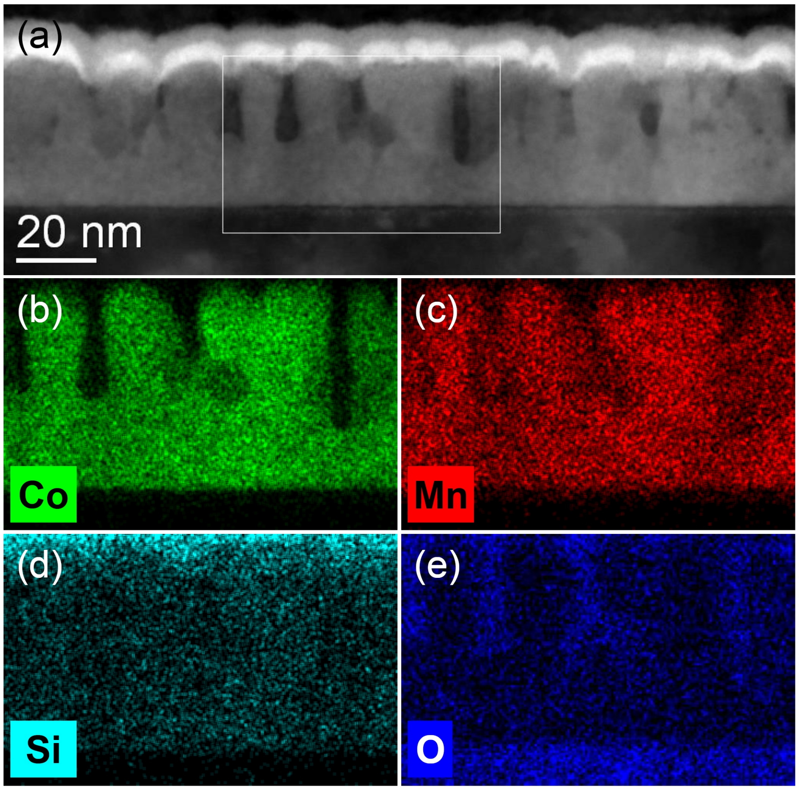

Figure 5. (Color online) Cross-sectional HAADF-STEM image of the sample irradiated with 5×10^{14} cm^{-2} He^{+} ions (a) together with the (b) cobalt, (c) manganese, (d) silicon, and (e) oxygen element distributions obtained by EDXS spectrum imaging for the area marked with the white rectangle in part (a).

NMR spectra analysis

Figs. 6(A)-(F)(a) show the same Gaussian fits to the spectra as discussed in the main text for the reference sample up to the sample irradiated with 1×10^{15} ions/cm^2. The corresponding fluence is written in the graphs. The Gaussians describing the $L2_1$ phase of one particular sample are of equal width and equidistant from each other, reflecting the symmetry of the local field. (The equidistance results from the fact that the change of the ^{59}Co hyperfine field upon replacing two nearest neighboring (nn) Mn with two Co atoms is twice as large as the change upon replacing one nn Mn with one Co atom. The FWHM is defined by disorder in higher shells, which stastically is the same for all ^{59}Co nuclei.) An amazing agreement is found for the off-stoichiometry of the $L2_1$ phase. For all spectra, the $L2_1$-ordered part can be well described with a Co^{Mn} antisite concentration of $x = 7.25$ %, as can be seen in the corresponding column diagrams (b) and the difference between the binomial distribution function and the Gaussian areas (c), not exceeding 4 % even for the highest fluences.

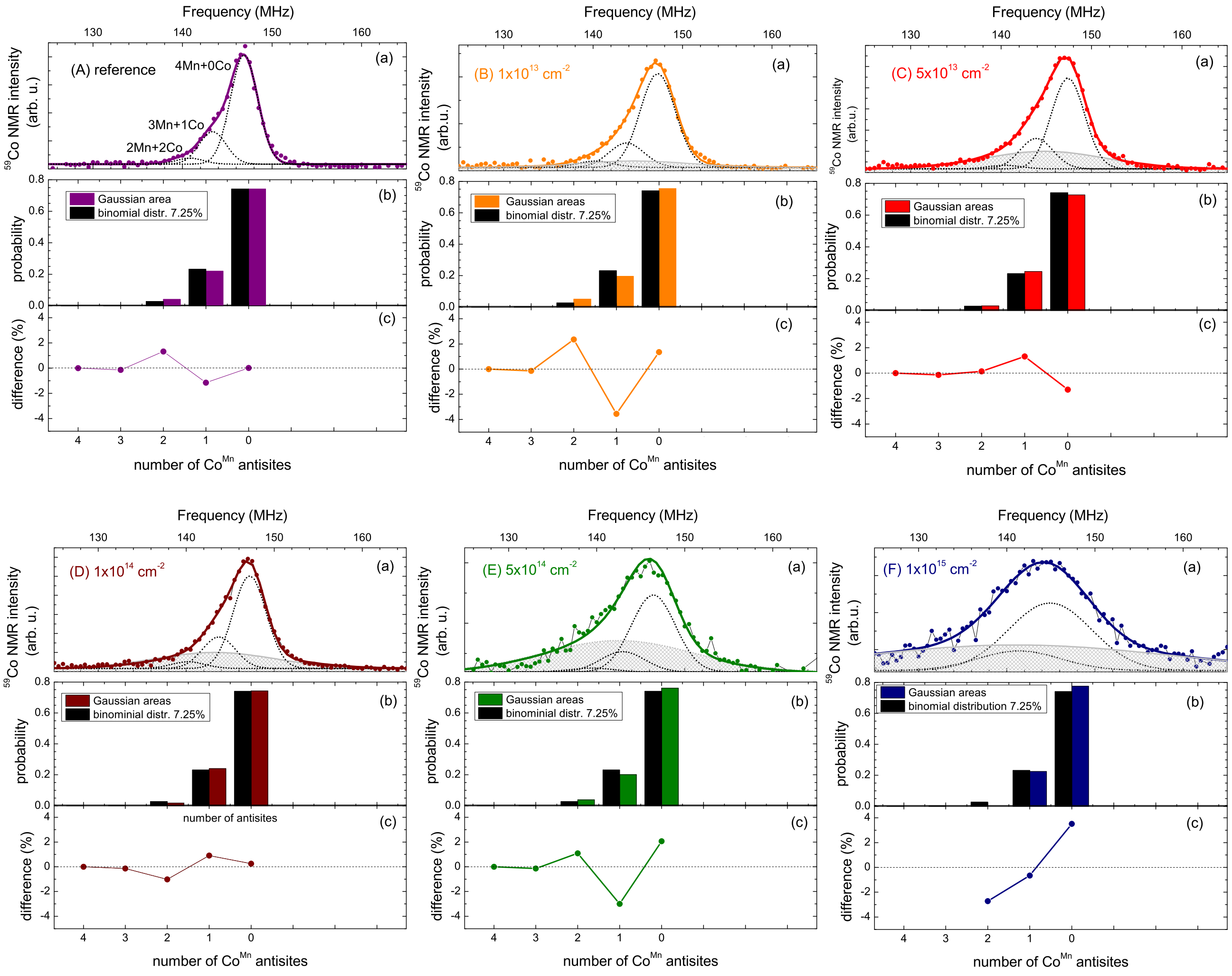

Figure 6. (Color online) (a) ^{59}Co NMR spectra at 5 K for all samples from the non-irradiated one up to an irradiation of 1×10^{15} ions/cm^2. The corresponding fluence is written in the graphs. Lines are fits with Gaussian lines to the spectra. The black dotted lines denote the Gaussians representing the off-stoichiometric $L2_1$ ordered regions of the samples. The text next to these Gaussians in panel (A) denote the corresponding first coordination shell environment of ^{59}Co. The filled light grey line starting panel (B) corresponds to $A2$-ordered regions of the sample. The thick line in the color of the respective data dots is the sum of all Gaussian lines. (b) Probability $P(n,x)$ of the binomial distribution function for $x = 7.25\,\%$ (black columns) in comparison with the relative areas of the grey Gaussian fits to the spectra [column color same as for the corresponding data points in (a)]. (c) Differences between the relative areas of the grey Gaussian lines and the binomial probability.

References

1. Rodan, S. *et al.* Nuclear magnetic resonance reveals structural evolution upon annealing in epitaxial Co_2MnSi Heusler films. *Appl. Phys. Lett.* **102**, 242404 (2013).
2. Wurmehl, S. & Kohlhepp, J. T. NMR spectroscopy on Heusler thin films - a review. *Spin* **4**, 1440019 (2014).
3. Wurmehl, S. *Structural Order in Heusler Compounds. In: Heusler alloys - properties, growth, applications.* (Springer, 2016). (Eds. C. Felser, and A. Hirohata), and references therein.
4. Bacon, G. E. & Plant, J. S. *J. Phys. F: Met. Phys.* **1**, 524 (1971).
5. Inomata, K. *et al. J. Phys. D: Appl. Phys.* **39**, 816 (2006).
6. Inomata, K., Wojcik, M., Jedryka, E., Ikeda, N. & Tezuka, N. *Phys. Rev. B* **77**, 214425 (2008).
7. Wurmehl, S. *et al. Appl. Phys. Lett.* **98**, 12506 (2011).
